# High accuracy capillary network representation in digital rock reveals permeability scaling functions

**DOI:** 10.1038/s41598-021-90090-0

**Published:** 2021-06-15

**Authors:** Rodrigo F. Neumann, Mariane Barsi-Andreeta, Everton Lucas-Oliveira, Hugo Barbalho, Willian A. Trevizan, Tito J. Bonagamba, Mathias B. Steiner

**Affiliations:** 1 IBM Research, Rio de Janeiro, RJ 22290-240 Brazil; 2 São Carlos Institute of Physics, University of São Paulo, PO Box 369, São Carlos, SP 13560-970 Brazil; 3 CENPES/Petrobras, Rio de Janeiro, RJ 21941-915 Brazil; 4 Present Address: Dell Technologies, Rio de Janeiro, RJ 21941-907 Brazil

**Keywords:** Applied physics, Fluid dynamics, Applied mathematics, Scientific data, Software, Petrology, Porous materials

## Abstract

Permeability is the key parameter for quantifying fluid flow in porous rocks. Knowledge of the spatial distribution of the connected pore space allows, in principle, to predict the permeability of a rock sample. However, limitations in feature resolution and approximations at microscopic scales have so far precluded systematic upscaling of permeability predictions. Here, we report fluid flow simulations in pore-scale network representations designed to overcome such limitations. We present a novel capillary network representation with an enhanced level of spatial detail at microscale. We find that ﻿the network-based flow simulations predict experimental permeabilities measured at lab scale in the same rock sample without the need for calibration or correction. By applying the method to a broader class of representative geological samples, with permeability values covering two orders of magnitude, we obtain scaling relationships that reveal how mesoscale permeability emerges from microscopic capillary diameter and fluid velocity distributions.

## Introduction

**Figure 1:**
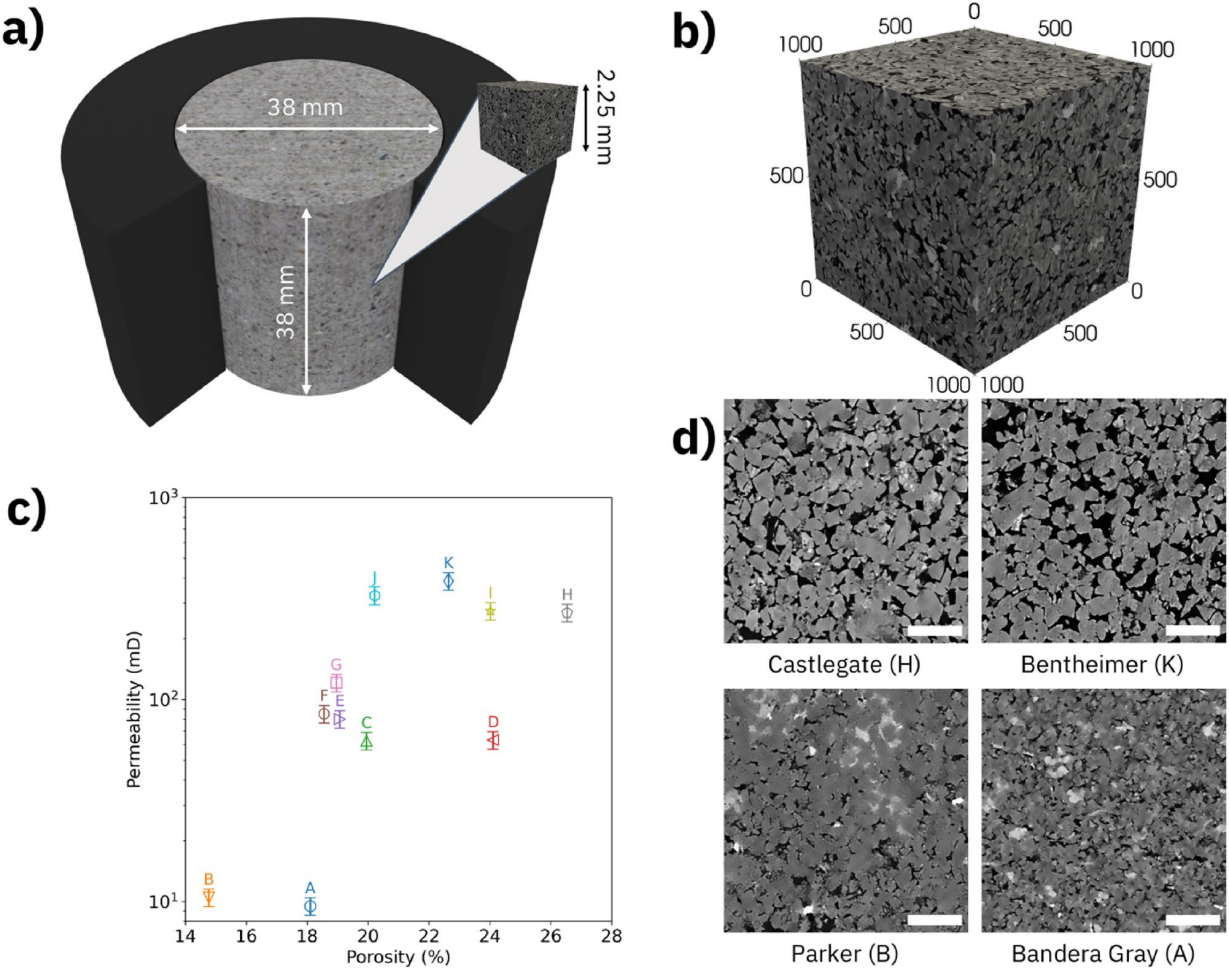
Conception of permeability and porosity determination in rock samples. (**a**) Permeability and porosity measurements are performed at lab scale. However, computational simulations are performed with microscopic network representation at pore scale. 3D visualization created using Blender v2.92.0 software. (**b**) Pore scale representation of a rock sample derived from an X-ray microtomography with 1000 voxels of 2.25 µm along each side, resulting in an overall side length of 2.25 mm. 3D visualization created using ParaView v5.9.0 software. (**c**) The experimental porosity–permeability plot of containing all rock samples. (**d**) Representative grayscale images extracted from the data cube of the least porous (B), least permeable (A), most porous (H) and most permeable (K) rock sample, respectively, exhibit the microstructural variation occurring at pore scale. The white scale bar at the lower right corner of each image represents 500 µm.

Permeability is a critical figure-of-merit in the characterization of porous geological samples for applications ranging from water management to fluid recovery and carbon dioxide sequestration^1–8^. Fluid flow assessment in rocks typically involves multiple physical length scales; a fluid passes through a complex, interconnected network of capillaries with diameters ranging from nanometers to millimeters, while flow conditions are typically set and measured at lab scale, see Fig. 1. Once the spatial distribution of the connected pore space in a rock sample is known, a flow model is applied to computationally predict the fluid permeability based on the capillary network geometric boundaries^9–14^. We show in the following that a high-accuracy, network-based representation of a microscopic fraction of a rock’s connected pore space is a suitable template for computationally predicting experimental permeability results obtained from the same rock sample at lab scale, a volume upscaling by 3 orders of magnitude. An application of the method to 11 sandstone samples, see Table 1, reveals permeability scaling as function of diameter distribution and flow speed. We suggest that the extracted slopes be used more generally for characterizing permeability scaling in this geological sample class.

**Table 1:** List of sandstone rock samples used in our study and their respective properties.

Sample	Name	Porosity (%)	Permeability (mD)
A	Bandera Gray	18.10	9
B	Parker	14.77	10
C	Kirby	19.95	62
D	Bandera Brown	24.11	63
E	Berea Sister Gray	19.07	80
F	Berea Upper Gray	18.56	86
G	Berea	18.96	121
H	Castlegate	26.54	269
I	Buff Berea	24.02	275
J	Leopard	20.22	327
K	Bentheimer	22.64	386

Sample labels A–K are listed in increasing order of permeability. We estimate the experimental error to be ± 0.5% for porosity and ± 10% for permeability, respectively^15^.

Methodically, the application of X-ray microtomography to rock samples provides a series of images of the spatial distribution of the pore space from which three-dimensional digital rock representations are created^16–18^. Once a full series of microscopic rock images is acquired, the image series undergoes a sequence of processing steps with regards to noise, segmentation and morphology, producing a data cube (digital rock) containing voxels that either represent solid or void space of the imaged rock^19^. Here, the spatial discretization method—such as meshes and grids, used by Finite-Element, Finite-Volume and Lattice Boltzmann; or nodes and edges, used by network-based methods—depends on the flow simulation algorithm to be used for performing subsequent permeability predictions^20–25^. Mesh and grid-based numerical methods have the advantage of being more adaptable—to within the size of the voxel—to the geometry of the pore space^17^. However, the requirement to fill the entire pore space with either mesh elements or grid points makes these methods less computationally efficient^20,25^. Network-based models, on the other hand, balance geometrical accuracy and computational complexity by representing pore space through geometrical primitives, e. g. cylinders and spheres, for which flow properties are typically obtained in (semi-) analytical form^14,20,24^.

In any of these methods, the tradeoff between the voxel (volume pixel) size and the total imaged sample volume poses practical limits to the spatial resolution of lab scale samples in which permeability measurements are typically performed. The computational representations of lab scale rock sample do not fully resolve the diameter distribution of the capillary network, which consequently leads to inaccuracies in permeability predictions. In addition, computational approximations of the connected pore space by geometrical primitives, such as balls and sticks, can be insufficient to capture the actual complexity of the capillary network at pore scale. Sample heterogeneities can further complicate the picture; the higher the spatial heterogeneity of a rock sample the larger the Representative Elementary Volume (REV) for quantifying the threshold sampling volume at which the statistical properties of the capillary network become representative for the entire lab scale rock sample^26–29^. In essence, a combination of stringent requirements needs to be met for successfully predicting the permeability of lab scale samples based on microscopic capillary network representations.

For establishing those requirements, we have systematically analyzed a representative group of highly resolved, three-dimensional microscopic image cubes measured on rock samples for which permeability and porosity have been experimentally verified at lab scale. For evaluating the influence of pore scale representation quality on the accuracy of permeability predictions, we have used three candidate network representations with varying levels of spatial detail as geometrical template: (1) the *Capillary Network Model* (CNM), which transforms the pore space into a voxel-wide line at the center of the pore channels; (2) the *Reduced Max Ball Model* (RMB)^30^, in which the network is constructed using connecting cylinders modelled by longer line segments that follow the medial axis of the pore space; and (3) the *Pore Network Model* (PNM)^31^, which divides pore space into pores and throats, where each pore is a node in the network and each throat is a link between nodes. For each sample, we have compared the computational permeability predictions obtained for each of the three pore space representations with the experimental permeability obtained from the same rock sample at lab scale. By aggregating the data of all samples studied, we have obtained the scaling relationships and slopes for characterizing more broadly the permeability of the entire sandstone sample class.

## Results

### Experimental characterization

We have measured the porosity and permeability of a set of 11 cylindrical sandstone samples with radii and heights of 19 and 38 mm, respectively. The connected porosities found in these samples range from 14 to 27%, with a mean of 20 ± 3%, and the absolute permeabilities from 9 to 386 mD, with a mean of 150 ± 40 mD, as summarized in Table 1. Details on the flow measurements are provided in the “Methods” Section. In Fig. 1c, we plot the measured gas permeability and porosity for each sample. The microscopic distributions of connected pore space in each rock are mainly responsible for the observed porosity/permeability variations as can be examined by visual inspection of the X-ray microtomography images of the samples. In Fig. 1d, we show representative images taken at the center of the image cube of the least porous (B), least permeable (A), most porous (H) and most permeable (K) rock samples in the dataset.

We have acquired X-ray microtomography image cubes on sub-samples (height = 30 mm, radius = 5 mm) extracted from each of the rock plugs listed in Table 1 (see “Methods” Section for details). The representative three-dimensional microtomography in Fig. 1b has a voxel size of 2.25 µm in which the lighter (darker) gray levels correspond to solid (void) space in the sandstone. For extracting the connected pore space, we have processed each raw image (gray scale) cube through a workflow that includes contrast-enhancement, noise reduction and threshold-based segmentation (see “Methods” Section for details). The resulting binary image cube contains the spatial map of pore space inside the sample. The volume fraction of voxels identified as pores is readily available, however, a subsequent processing step is needed to remove isolated pores that do not contribute to permeability.

We have then used the connected pore space in the image cube as a template for performing numerical flow simulations and permeability predictions. By analyzing the dependence of flow simulation accuracy on rock image cube size, see Supplementary Figure S2, we obtain REV = (2.25 mm)^3^. Finally, we obtain a volume scaling factor of V_exp_/REV = 3784, which relates the connected pore space volume used for computing permeability predictions to the reference sample volume that we have probed in our lab experiments.

### Numerical simulations

For quantifying the accuracy of geometric approximation in the digital rocks, we have compared three network-based representations of the microscopic pore space—PNM as algorithmically implemented in the PoreSpy open-source package^32^, RMB based on the algorithm developed by Andreeta et al*.*^30^ and CNM performed by an algorithm reported here for the first time. The particular choice of candidate network representations maps the spectrum of network models in terms of spatial detail, from PNM (coarse) via RMB to CNM (fine).

**Figure 2:**
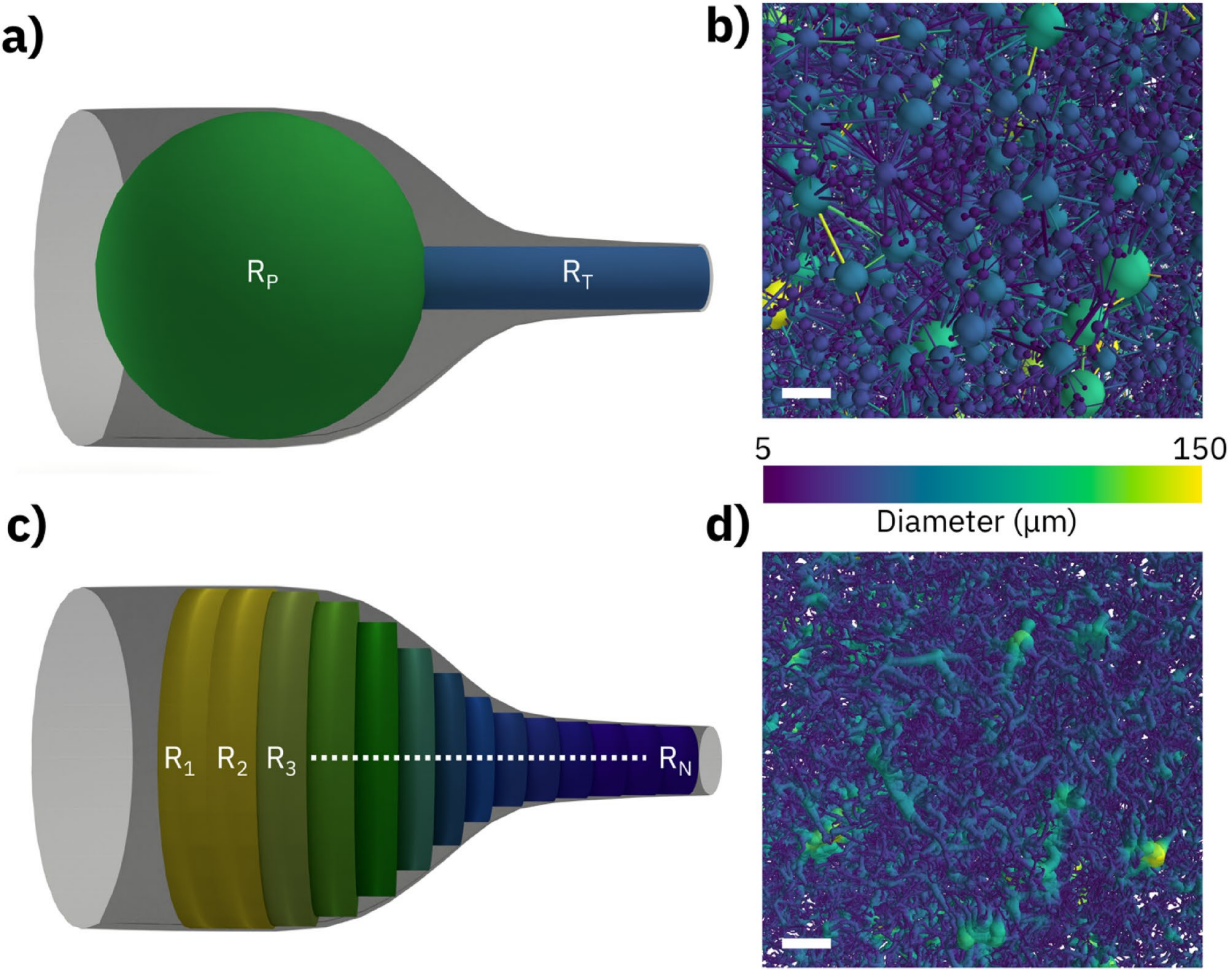
Visual conceptions (left) and implementation examples (right) of representative network models. (**a**) The Pore Network Model separates the pore space into spherical pores with radius R_P_ and cylindrical throats with radius R_T_. (**b**) Pore Network Model representation using color-coded diameters. (**c**) The capillary network model separates the pore space into a sequence of short cylinders with gradually changing radii {R_n_}. (**d**) Capillary Network Model representation using color-coded diameters. The white scale bar represents 100 µm. Visual conceptions (**a**,**c**) created using Blender v2.92.0 software.

In Fig. 2, we show pore network (top) and capillary network (bottom) representations applied to the same sample region. In a pore network representation, see Fig. 2a, the void space is sub-divided into pores and throats. Pore and throat are each represented by their radius, R_P_ and R_T_, respectively, while specifics of the cross-sectional geometries are matched using shape factors^14^. In Fig. 2b, a network of spheres and cylinders geometrically approximates the occurring void space. For comparison, Fig. 2c exemplifies how a capillary network representation would approximate the same sample region. Here, the void space is filled with short (one voxel long) cylinders having their radii {R_n_} evolving gradually to match the local boundaries. The resulting network of connected capillaries in this approximation is shown in Fig. 2d.

In PNM, nodes are associated with pores, which have a finite volume, and links are associated with throats, which impose a resistance to the flow between the two nodes it connects. In CNM, on the other hand, nodes are considered zero-volume points that do not contribute to the pore space, while links represent finite-volume cylinders that account for all the pore volume and flow resistance. Finally, RMB has features of both pore and capillary network representations as it uses extended cylinders to represent the pore space.

**Figure 3:**
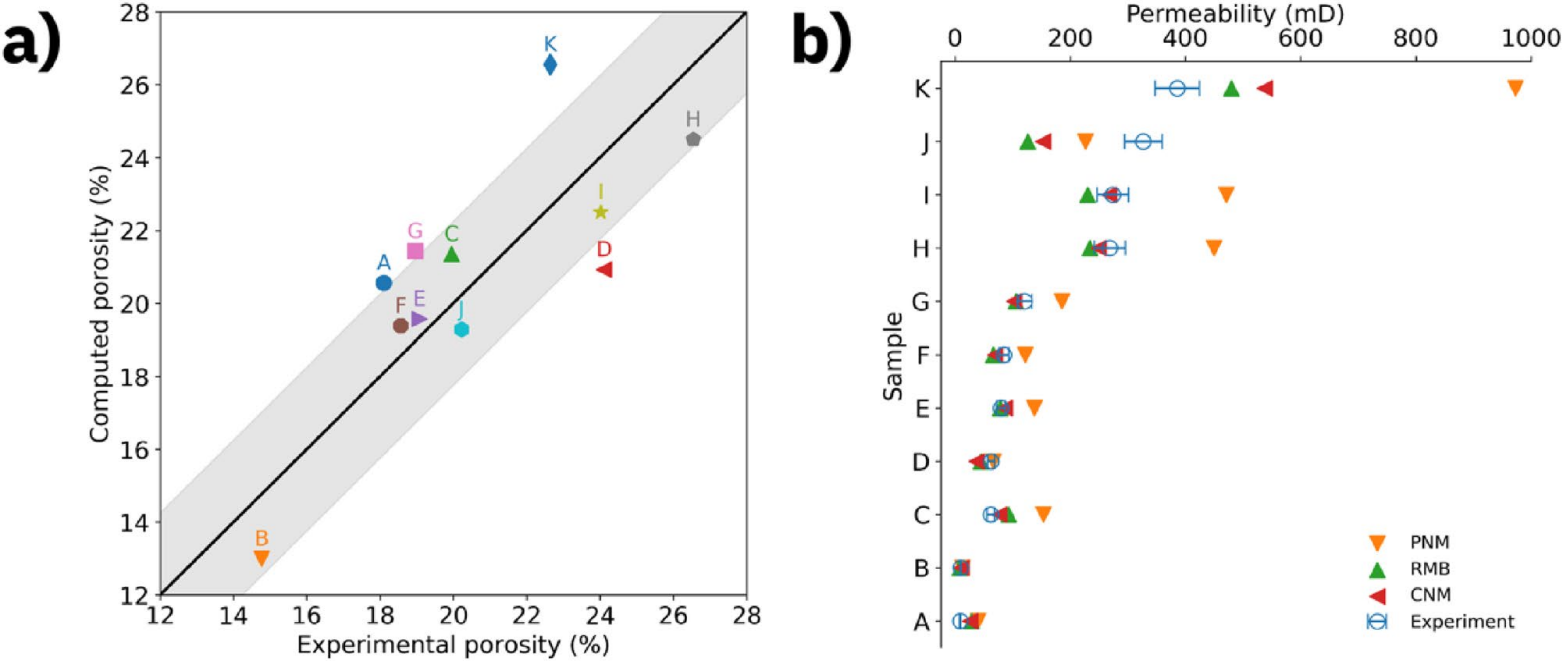
Comparison between experimental and computed porosities and permeabilities. (**a**) Computed versus experimental porosity for all samples studied. The solid line indicates agreement between computation and experiments and the gray shaded area represents the root mean squared error of 2.25 percentage points. (**b**) Experimental and simulated permeability for all samples studied. The computational results represent the mean permeability along the three main axes. Experimental results are represented by (blue) open circles, those from the Pore Network Model by (orange) filled down-pointing triangles, those from the Reduced Max Ball Model by (green) filled up-pointing triangles and those from the Capillary Network Model by (red) filled left-pointing triangles.

In Fig. 3, we have compared the experimental and computational porosities and permeabilities. The computational porosity values extracted from the connected pore space in digital rocks are plotted against the experimental porosity values in Fig. 3a. The diagonal indicating a perfect match between experiment and computational prediction is overlaid by a gray shaded area indicating a root mean squared error of 2.25%. Nine of eleven samples fall within the shaded area, indicating reasonable agreement.

In Fig. 3b, we plot the experimental and simulated permeabilities for each network model considered in this study. In each of the three network representations, we have simulated water flow assuming a 10 kPa/m pressure gradient across the sample. The flow simulation algorithm solves a linear system of equations that applies Poiseuille law at each network link (i.e., cylindrical capillary) and mass conservation law at each network node (for details see “Methods” Section). The simulated data points represent the quadratic mean of the simulated permeability along the three main axes. Considering the entire sandstone sample set, we observe the best agreement for CNM with a mean relative error between model prediction and experimental result of 38%, followed by RMB with 42%, attesting to the high accuracy of the geometrical approximation achieved with this approach. In contrast, the PNM based predictions are significantly less accurate, at a mean relative error of 92%, with a maximum mismatch of more than a factor of two in the case of sample K. In all cases, the agreement between simulated and experimental permeabilities decreased for higher permeability samples, as previously reported^25^.

**Figure 4:**
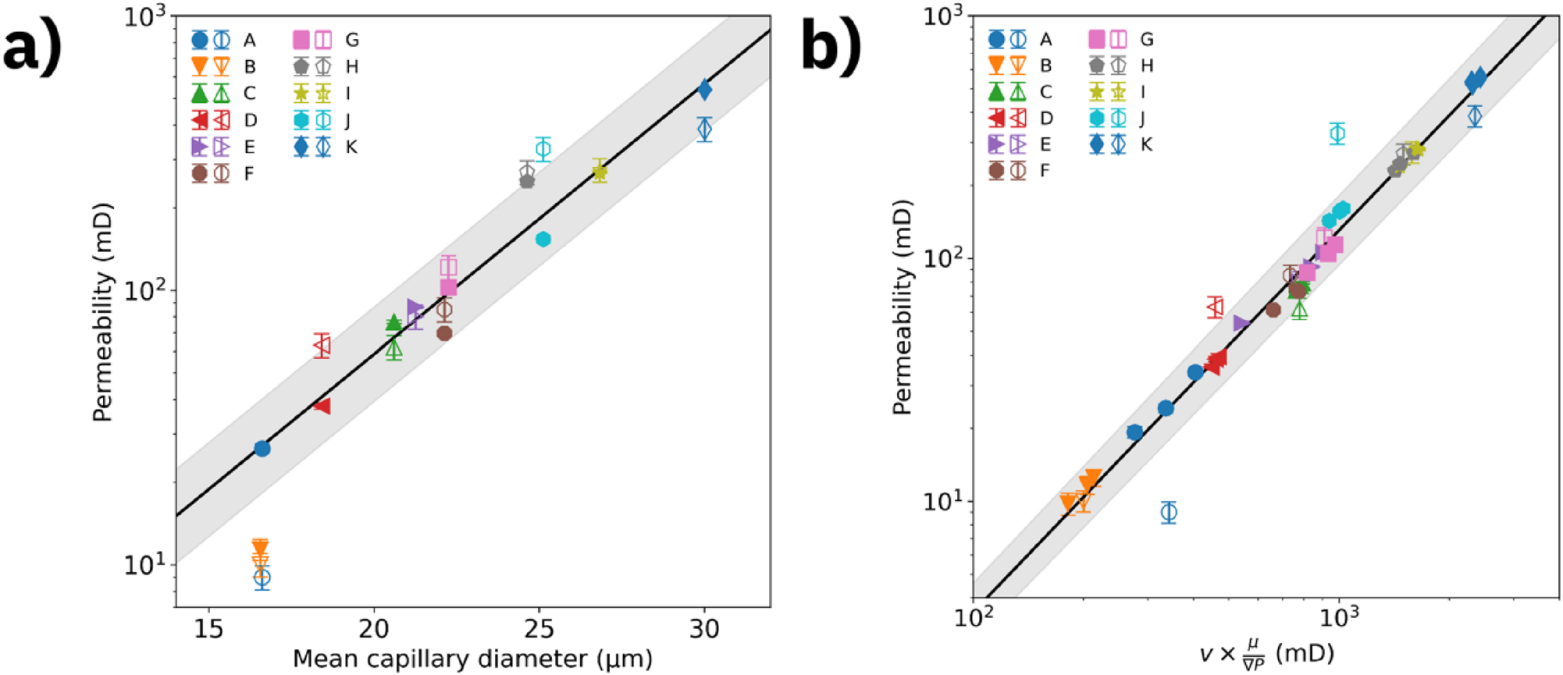
Scaling of permeability as function of mean capillary diameters and mean flow speeds. Computed (experimental) permeability is represented by filled (open) symbols. (**a**) Permeability as function of mean capillary diameter for all rock samples studied. (**b**) Permeability as function of volume-averaged flow speed multiplied by $\left( {\mu /\nabla P} \right)$ along all 3 axes for all rock samples studied. The plot aggregates results from simulation scenarios with viscosity µ = 1 cP and pressure gradient of 10 kPa/m, as well as variations with 10 × higher viscosity or 10 × stronger gradient, covering two orders of magnitude variation in flow speeds. The lines represent linear fits to the data and the shaded area represents the fit uncertainty. The computed data are CNM results.

As a key result of this investigation, we plot in Fig. 4, for each sample, the relationship between lab scale permeability and microscopic properties based on CNM. While bulk permeability and porosity of the sample class are broadly scattered, see Fig. 1, the permeability (both experimental and computed) scales with the mean capillary diameter observed in the samples, see Fig. 4a. A linear fit to the data provides a slope of (0.098 ± 0.007) D[µm] for $\log_{10} K\left[ {{\text{mD}}} \right]$, which characterizes the entire sandstone sample set. In Fig. 4b, we plot experimental and computed permeability as a function of generalized velocity $u = \left( {v \times \mu /\nabla P} \right)$, where $v$ is the (microscopic) volume-weighted average flow speed inside the capillaries, $\upmu$ is the dynamic viscosity of the fluid and $\nabla P$ is the pressure gradient along the flow axis. A linear fit to the data (R^2^ > 0.98) in a $\log_{10} K\left[ {{\text{mD}}} \right] \times \log_{10} u\left[ {{\text{mD}}} \right]$ plot of indicates that permeability is well-described by (0.0024 ± 0.0003) × u^1.57±0.02^ for the entire set of sandstone samples.

A practical application of the scaling results is estimating lab scale permeabilities based on mean capillary diameters and flow speeds taken from CNM predictions performed on X-ray microtomography data or, conversely, getting insight into microscopic flow speeds based on lab scale permeability measurements.

## Discussion

We have computationally predicted lab scale permeabilities based on X-ray microtomography data for a sample set containing 11 sandstones. For performing fluid flow simulations, we have used three network-based geometrical representations of a microscopic volume fraction of the connected pore space of a rock. The representation with the highest level of spatial detail, CNM, enables predicting experimental permeability results obtained from the same rock sample at lab scale (more than 3000 × volume) with a mean relative error between microscopic model prediction and lab scale experimental result of 38%. By aggregating the results, we have obtained scaling relationships between the lab scale permeabilities and the mean capillary diameter and flow speed, respectively, for the sandstone sample class that can be used for estimating lab scale permeabilities based on X-ray microtomography data. These results demonstrate the relationship between accuracy of microscopic detail employed by the network representation and the scaling potential of the ensuing permeability calculation. Future research should extend the above methodology to geological sample classes with higher degrees of heterogeneity and more complex capillary diameter distributions, such as carbonate and shale rocks. The extension of the work to these rock types will likely require smaller voxels and larger sampling volumes, which is expected to increase the computational cost of network-based methods, but still less than the expected cost increase for mesh- and grid-based methods.

## Materials and methods

### Rock samples

We have acquired a set of sandstone rocks (*Kocurek Industries INC*.) with the following 11 samples: Bandera Gray (A), Parker (B), Kirby (C), Bandera Brown (D), Berea Sister Gray (E), Berea Upper Gray (F), Berea (G), Castlegate (H), Buff Berea (I), Leopard (J) and Bentheimer (K).

### Lab permeability measurement

We have experimentally characterized the cylindrical plug samples (height = 38 mm, radius = 19 mm) at lab scale following the API RP-40 norm^33^. In a first step, we have measured porosity through Helium pressure variations in a Boyle's Law Double Cell. In a second step, we have measured permeability by monitoring steady-state Nitrogen flow rate in the axial direction of the cylindrical samples^34^. We have applied an axial pressure gradient of 30–40 psi to the samples for establishing laminar flow conditions. For restricting fluid flow to the axial direction, we have confined the plugs laterally by applying a radial pressure through a rubber sealing. We have limited the radial pressure to 500 psi so to avoid significant modifications of the plug’s pore volume. Finally, we have applied a Klinkenberg correction^35^ to correct for fluid expansion. We estimate the experimental error to be ± 0.5% for porosity and ± 10% for permeability, respectively^15^.

### X-ray microtomography

In a next step, we have sub-sampled the plugs to obtain smaller cylindrical samples (height = 30 mm, radius = 5 mm). We have obtained pore scale image data by using high-resolution 3D X-ray Microtomography (SkyScan 1272, Bruker). We have set source voltage and current to 50 kV and 200 µA, respectively. We have configured the CCD camera to acquire projections of 4904 × 3280 pixels, resulting in a pixel side length of 2.25 µm. We have performed image reconstruction using SkyScan NRecon (version: 1.7.0.4, Bruker).

### Image processing

We have processed the rock image data into subsets containing 1000^3^ cubic voxels and converted the image from 16-bit to 8-bit gray scale. We have then applied an enhancement filter to equalize the contrast across multiple images. For each data set, we have cut off the grayscale level where the accumulated grayscale histogram achieved 99.8% and mapped the remaining grayscale levels to the [0, 255] interval. To reduce image noise, we have executed on each data set a 3D non-local means filter^36,37^ available in Fiji^38^ using a smoothing factor of 1 and automatically estimated sigma^39^ parameters. Finally, by using a threshold level calculated by the IsoData method^40^, we have segmented the noise-reduced grayscale images into solid and void space leading to a binary image. The image-processing parameters for each sample are available in Supplementary Table S1. We have then processed the binary images using the Enhanced Hoshen-Kopelman algorithm^41^ for morphological analysis and, in a final step, eliminated from each data cube the pore voxels that are not connected to the percolating network.

### Network extraction

We have then used the binary image data sets containing the connected pore voxels as input to three representative network extraction algorithms: PNM, RMB and CNM. Briefly, all algorithms calculate distance maps of the images and construct a hierarchical graph of the voxels associated with the void space.

The PNM is extracted using the SNOW algorithm based on watershed transformation^31^, available in PoreSpy^32^ version 1.2.0, which is specialized in extracting pore networks from high-porosity materials.

The RMB algorithm^30^ is a modified medial-axis extraction based on the original Max Ball Algorithm. The pore-throat connections in the Max Ball Algorithm are found through the maximum ball chains. These chains are further processed in the RMB by finding the optimum fluid flow paths between pore centers through Dijkstra’s algorithm. The final output is a simplified medial axis composed by the spheres on the optimum paths’ chains. The sphere´s centers are the nodes in the network and the links are modelled by the neighboring spheres radii and distance between centers.

The CNM representation is based on the *Centerline* algorithm^42^. A centerline is a thin, one-dimensional object that captures a 3D object’s main symmetry axes, summarizing its main shape into a set of curves^43^. In this work, we have used for the first time an algorithm that extracts the centerlines of a 3D image by using an adaptation of the traditional Dijkstra’s Minimum Path algorithm in a graph with penalized distance. The full algorithm description can be found in the Supplementary Information.

### Flow simulation

The flow simulation algorithm applied in this study operates on nodes and links of the network representations. Specifically, the algorithm applies Poiseuille law to the links and mass conservation law to the interior nodes, while maintaining a fixed pressure difference between inlet and outlet boundary nodes. This is represented by a system of mass conservation equations Σ_j_ Q_ij_ = 0 for all nodes i, where Q_ij_ = (πR_ij_^4^/8µL_ij_) (P_i_ − P_j_) is the flow rate in the capillary that connects node i to node j. The geometrical parameters R and L, respectively, represent the radius and the length of a capillary (link) connecting two nodes of the network and µ is the dynamic viscosity of the fluid. Unless stated otherwise, all simulations used a viscosity of µ = 1 cP and applied a 10 kPa/m pressure gradient along the flow direction in addition to atmospheric pressure. We have calculated permeability using Darcy’s Law Q = K (A/µL) ΔP, which causes µ and ΔP to factor out and not affect the results. In order to validate the flow simulations, we have benchmarked them against OpenPNM^44^ version 2.4.2, with results matching within ± 1 mD.

## Supplementary information

Supplementary Information.

## Data Availability

The microtomography datasets generated and/or analyzed during the current study are available in the Digital Rocks Portal repository, at https://dx.doi.org/10.17612/f4h1-w124.

## Code availability

The code used to obtain the PNM results reported in the current study are available in a Github repository, at https://github.com/mbandreeta/absolute_permeability_simulations.
