# Supplementary material for: High accuracy capillary network representation in digital rock reveals permeability scaling functions
Source: Sci Rep. 2021 Jun 15;11:11370. doi: 10.1038/s41598-021-90090-0 (PMC8206086; doi:10.1038/s41598-021-90090-0)
Supplement: Supplementary file 1 — Supplementary Information. [file 41598_2021_90090_MOESM1_ESM.pdf]

High accuracy capillary network representation in digital rock reveals permeability scaling functions

Rodrigo F. Neumann[1], Mariane Barsi-Andreeta[2], Everton Lucas-Oliveira[2], Hugo Barbalho[1†], Willian A. Trevizan[3], Tito J. Bonagamba[2] and Mathias Steiner[1*]*

[1] IBM Research, Rio de Janeiro, RJ, 22290-240, Brazil
[2] São Carlos Institute of Physics, University of São Paulo, PO Box 369, São Carlos, SP, 13560-970, Brazil
[3] CENPES/Petrobras, Rio de Janeiro, RJ, 21941-915, Brazil
[†] Present address: Dell EMC R&D Center, Rio de Janeiro, RJ, 21941-907, Brazil
[*] Corresponding authors: rneumann@br.ibm.com and mathiast@br.ibm.com

Supplementary Information

Image-processing method parameters

Supplementary Table S1: Image-processing parameters for rock image cubes. Prior to segmentation, the contrast enhancement filter cut off the grayscale histogram at the described cut-off level and rescaled all grayscale levels to [0, 255]. Finally, after running the non-local means filter, the grayscale image was segmented using a threshold level calculated by the IsoData algorithm.

Sample	Name	Cut-off level	Threshold level
A	Bandera Gray	93	81
B	Parker	63	72
C	Kirby	61	75
D	Bandera Brown	73	80
E	Berea Sister Gray	90	54
F	Berea Upper Gray	101	48
G	Berea	71	59
H	Castlegate	156	81
I	Buff Berea	62	73
J	Leopard	76	72
K	Bentheimer	200	71

Computed porosity and permeability

Supplementary Table S2: Experimental and computed results for porosity and permeability. Porosity results compare experimental measurements to values computed from microtomography data. Permeability results compare experimental values to Pore Network Model (PNM), Reduced Max Ball Model (RMB) and Capillary Network Model (CNM) values. We estimate the experimental error to be ±0.5% for porosity and ±10% for permeability, respectively [15].

Sample	Name	Porosity (%)		Permeability (mD)			
		Exp.	µCT	Exp.	PNM	RMB	CNM
A	Bandera Gray	18.10	20.56	9	40	29	26
B	Parker	14.77	13.00	10	13	8	11
C	Kirby	19.95	21.35	62	154	93	76
D	Bandera Brown	24.11	20.93	63	67	45	38
E	Berea Sister Gray	19.07	19.57	80	138	79	86
F	Berea Upper Gray	18.56	19.38	85	122	66	70
G	Berea	18.96	21.43	121	186	106	102
H	Castlegate	26.54	24.50	269	450	234	249

I	Buff Berea	24.02	22.50	274	471	230	267
J	Leopard	20.22	19.28	327	226	126	153
K	Bentheimer	22.64	26.56	386	973	480	538

Capillary network extraction algorithm

A commonly used formal definition of the centerline, which is agnostic to the extraction algorithm, requires these four properties to be obeyed:

1. **Connected:** There must have at least one path between any two voxels of the centerline.
2. **Centered**: Any voxel of the centerline must be centered with respect to the object's boundary.
3. **Thin**: Centerline should be only 1-voxel thick.
4. **Insensitive to boundary noise:** Small surface details should not produce large twists or numerous small branches in the centerline.

In the following, we outline a novel adaptation of the Dijkstra shortest path algorithm to extract the centerline, or CNM, from a rock tomography image cube. The algorithm uses a penalization function and gradient vector for building a centerline with minimal centerline property violation, while detecting and connecting image voxels that form cycles in the image.

The main procedure of our algorithm is detailed in Algorithm 1. A graph *G(V, A)* is given to this algorithm representing the rock. The set *V* denotes the voxels *v* and *A* denotes the set of directed arcs (v_{out}, v_{in}) that leaves a vertex v_{out} and arrives at neighbor voxel v_{in}. On top of G, in line 1, every voxel *v* in V has its distance to the closest boundary of the rock computed and the weight of all arcs arriving at *v* set accordingly to its distance. Then, in line 2, the source voxels located at the rock's face are identified. These voxels will be used by the shortest path algorithm in the next steps. Subsequently, an empty set with all centerlines and an empty set of voxels visited by a centerline are initialized in lines 3 and 4, respectively. The loop that starts at line 5 iterates over the source voxels of the facet and, if a voxel was not yet visited by any other centerline of a previous iteration, the adaptation of the Dijkstra's algorithm is performed in line 7. The centerline in represented by a subgraph of *G*, namely $D_{centerline}$. Next, in line 8, the voxels in centerline $D_{centerline}$, denoted by V($D_{centerline}$), are marked as visited. In line 9, the computed centerline is stored in the set *all_centerlines*. Throughout the rest of this section, a detailed explanation of the methods in lines 1, 2, and 7 will be provided.

Algorithm 1: Centerline-Extraction (Graph: *G (V, A)*)

1 *distances, local_maxima* = Compute-Distance-Map(*G*)
2 *source_voxels* = Identify-Source-Voxels(*G*, *distances*)
3 *all_centerlines* = { }
4 *visited_voxels* = { }
5 For each *voxel* ∈ source_*voxels*:
6   If *voxel* ∉ *visited_voxels*:
7 $D_{centerline}$ = Compute-Centerlines(*G*, *distances*, source = *voxel,* targets= source_*voxels, local_maxima*)
8   *visited_voxels = visited_voxels* U V($D_{centerline}$)
9   *all_centerlines = all_centerlines* U $D_{centerline}$
10 return *all_centerlines*

In the first method, the algorithm establishes the distance map of the object by computing for each pore space voxel its closest distance to the boundary. The distance is determined by the recursive function $d_{min}(v)$ that calculates for a given voxel *v* the distance from *v* to the closest boundary voxel.

The boundaries voxels v_b are set as the base case of the recursion; $d_{min}(v_b) = 0$. For all other voxels v, their distance from the closest boundary voxel is defined as $d_{min}(v) = \min_{t \in N(v)} \{d_{min}(t) + E(v,t)\}$, where $N(v)$ is the set of v's neighbor voxels and $E(v,t)$ is the Euclidian distance between v and t. In the definition of neighborhood applied here, voxels v and t are neighbors if they share a Face, Edge or Vertex, leading to Euclidian distances of 1, $\sqrt{2}$ or $\sqrt{3}$, respectively.

The algorithm uses a priority list as its main data structure for iteratively computing $d_{min}(v)$ for every voxel v. First, boundary voxels v_b are added to the list with their priority set to 0. Then, in the main loop, the voxel t with lowest priority (voxel index is used as tiebreaker) is picked from the list and the distance to its neighbor voxel v (that were not yet picked from the list) is set as follow:

- $d_{min}(v) = d_{min}(t) + E(v,t)$ and included into the list, if visited for the first time; or
- $d_{min}(v) = \min\{d_{min}(t) + E(v,t), d_{min}(v)\}$, if already visited.

The process finishes once the priority list is empty. In addition, voxels v for which $d_{min}(v)$ is greater or equal to d_{min} with regards to all adjacent voxels are annotated as local maxima leading to the creation of labeled, local voxel clusters. The main differentiator of our algorithm with regards to published works [42, SR1, SR2] is that the source and targets voxels of the centerline to be used in the Dijkstra shortest path algorithm are placed in the face of the cube that represents the image.

Algorithm 2: Compute-Distance-Map (Graph: *G (V, A)*)

```
priority_list = { }
finished = { }
distances = { }
For every boudary voxel v:
   priority_list.add(v,0)
While priority_list not empty:
   v, dist = priority_list.pop_lowest_priority()
   finished.add(v)
   For v_n ∈ neighbors(v) – finished: # all neighbors except those already set as finished
      If v_n ∉ distances or dist + E(v,v_n) < distances[v_n]:
         distances[v_n] = dist + E(v,v_n)
         priority_list.add_or_update(v_n , distances[v_n])
local_maxima = { }
For every v ∈ V(G):
    If distance[v] >= max{distance[v_n] for v_n in neighbours(v)}:
        local_maxima.add(v)
return distances, local_maxima
```

For identifying those source voxels, a depth-first search (DFS) algorithm [SR3] is executed for every voxel residing in the faces of the cube. The DFS algorithm will run multiple times, once for each cluster of connected voxels in the face. We visualize the outcome of DFS application in Supplementary Figure S1, in which the voxels with the highest values of $d_{min}(v)$ in a cluster (in black) are selected and highlighted (in red).

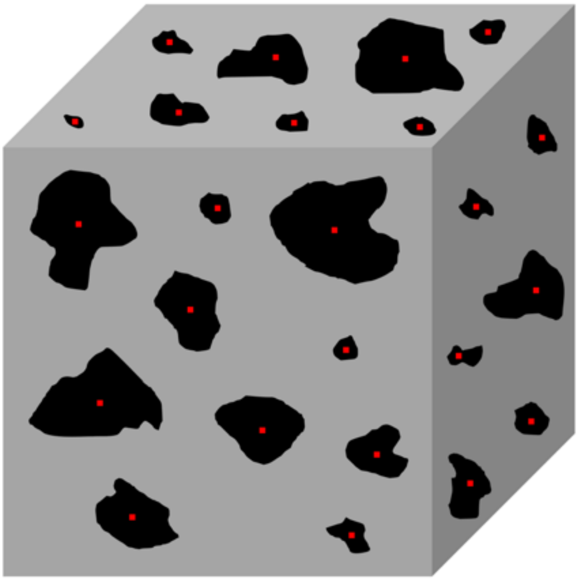

Supplementary Figure S1: Diagram of the rock sample tomography showing the rock (solid) voxels in gray and porous (void) voxels in black. For each face, a depth-first search algorithm identifies the most central voxel in each cluster (depicted in red). These voxels are then used as the source and target voxels for the Dijkstra shortest path algorithm. Simple diagram created using Microsoft® PowerPoint for Mac v16.46 software.

Algorithm 3: Identify-Source-Voxels (Graph: *G (V,A*), distances)

```
visited = { }
source_voxels = { }
For v ∈ V(G):
  If v ∉ visited and v is at a cube face:
    visited_dfs = DFS(G, v) # returns the set of face voxels visited in DFS starting at voxel v
    source = argmax{ distances[t] | t ∈ visited_dfs } # visited voxel in DFS with maximum distance
    source_voxels = source_voxels U {source}
    visited = visited U visited_dfs
return source_voxels
```

Once the process is completed for all faces, one of the selected voxels is set as source voxel and the others are considered target voxels for application of the Dijkstra shortest path algorithm [SR4]. The output of this algorithm is an acyclic connected graph (tree) consisting of pathways that connect the source voxel to target voxels. The centerline is then built by taking the subset of these pathways that connects source voxel to the target voxels.

In the following, we briefly outline the Dijkstra shortest path algorithm for computing the pathways and the penalization function for minimizing a centerline's property violations. The algorithm relies on two auxiliary data structures (see Algorithm 4): one that stores the cost of shortest path from the source voxel s to each voxel v; $c_{min}(v)$; and another one that stores the predecessor voxel of each voxel v; *pred*(v), associated with its respective shortest path to the source voxel. The cost function $c_{min}(v)$ in the original Dijkstra shortest path algorithm is accumulative, that is, it represents the cost of the entire path starting from the source voxel to the target voxel v. To compute the centerline, the Dijkstra shortest path algorithm version adapted here only accumulates the penalty cost from the previous

step. As a result, the algorithm connects voxel *v* to the neighbor that better suits the definition of the centerline by being “most centered” rather than the “shortest” path.

Similar to the original version of the algorithm, we use a priority list to iteratively compute the path from voxel *s* to each target voxel *v* that best fits the centerline definition. In each iteration, after voxel *t* is picked up from the priority list, the cost of the path of each of its neighbor voxel *v* that were not yet picked up from the priority list is updated as follow:

- $c_{min}(v) = penalty(t,v)$ and included into the list, if visited for the first time; or
- $c_{min}(v) = \min\{1 + penalty(pred(t), t) + penalty(t,v) + (1/ d_{min}(v)) * 1E3, c_{min}(v)\}$, if already visited.

In the first case, in which a known path from source to *v* does not exist, the path cost to traverse the graph from source to voxel *v* and the predecessor voxel of *v* are updated (*pred(v) = t*). In the second case, in which a path from the source to *v* is known, the path cost and the predecessor voxel are updated if, according to the *penalty* function, the connection between voxel *t* and *v* better represents the centerline than the existing connection between *pred(v)* and *v*.

The *penalty* function is vital for the algorithm to heuristically produce pathways matching the centerline definition as close as possible. For avoiding image boundaries, the *penalty* function takes into account a normalized gradient vector computed for every voxel based on distance transformation. The gradient vectors indicate which direction keeps the centerline away from the boundaries. The penalty of going from *t* to *v* depends on the angle α formed by the line that connects voxel *t* to *v* and the gradient vector at voxel *t*; $penalty(t,v) = 0.5 + (\sin^2(\alpha)+1) / d_{min}(v)$. However, inside a cluster of voxels annotated as local maximum, we use an alternative *penalty* function that sets a straight pathway.

Finally, we note that typical 3D rock tomography images contain centerlines with cycles. However, the centerline produced by the algorithm described above is acyclic. Therefore, we detect during the centerline algorithm potential pairs of voxels forming centerline cycles. A pair of neighbor voxels *t* and *v* is set to form a potential cycle when the voxel *v* is removed from the priority list has its neighbor *t* that was already removed from the queue and satisfies the following conditions: (1) $\sin^2(\alpha) < 0.1$, where *α* is the angle between the line from *t* to *v* and the gradient vector of *v* and, (2), the resultant cycle forms an *LMpath* [SR2].

Algorithm 4: Compute-Centerlines (Graph: *G (V, A)*, d_{min}, V: source, V: targets, local_maxima)

```
priority_list = { }
finished = { }
c_min = { }
pred = { }
end_points = targets
priority_list.add(source,0)
While priority_list not empty:
   t, dist = priority_list.pop_lowest_priority()
   finished.add(v)
   For v ∈ neighbors(v) – finished: # all neighbors except those already set as finished
      If  v  ∉ c_min:
         c_min[v] = penalty(t, v)
         priority_list.add(v , c_min[v])
         pred[v] = t
      Else:
```

```
            dist = 1 + penalty(pred[t], t) + penalty(t, v) + (1/ d_min (v)) * 1E3
            If dist < c_min[v]:
               c_min[v] = dist
               priority_list.update(v , c_min[v])
               pred[v] = t

      # Finding new end points
      For v ∈ neighbors(v) ∩ finished:
         α = gradient(t,v)
         If sin(α) < 0.1and LMpath(t, v, pred, local_maxima):
            end_points = end_points U { t, v}
   centerlines = build_centerlines(end_points, pred)
   return centerlines
```

REV determination

For obtaining the Representative Elementary Volume, we have simulated permeabilities for Berea (G) sample volumes with varying number of voxels in the cube, while keeping the voxel resolution at 2.25 µm. Specifically, we have analyzed 1000 samples with 100^3 voxels, 512 samples with 125^3 voxels, 125 samples with 200^3 voxels, 64 samples with 250^3 voxels, 8 samples with 500^3 voxels, and 5 samples with 1000^3 voxels. In Supplementary Figure S2, we show the resulting simulated permeability values as function of cube side length. We observe that the simulated permeabilities converge towards the experimental value. For a sample size of 1000 voxels (L = 2250 µm), the mean simulated permeability value matches the experimental permeability.

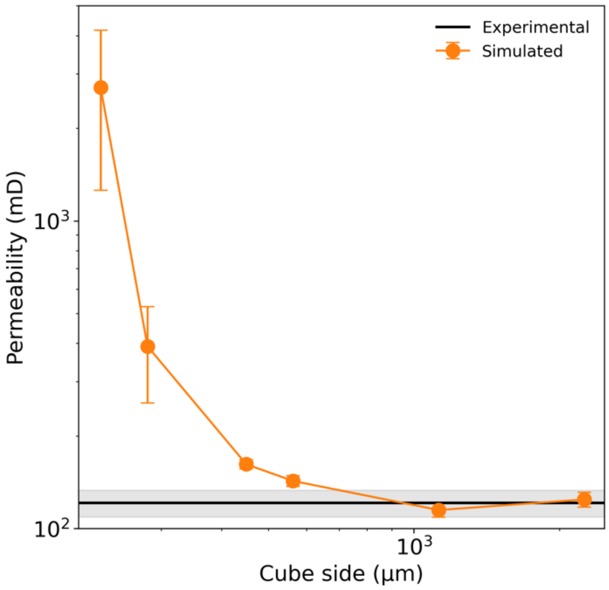

Supplementary Figure S2: Mean simulated permeability for Berea (G) as function of sample size. Error bars represent the standard error of the mean. The horizontal line indicates the measured permeability and the shaded area the experimental uncertainty. A match is achieved at a cube size of 2250 µm.

Distribution of capillary diameters and microscopic flow speeds

We have investigated the capillary diameter distributions of two representative samples in the set. Supplementary Figures S3(a) and S3(c) show the Parker (B) and Bentheimer (K) samples, respectively. Both Parker and Bentheimer exhibit a peak at around 10-15 µm in their capillary diameter distributions. However, Bentheimer distribution is broader with higher mean diameter of 30 µm, as compared to 16 µm for Parker.

To analyze the flow behavior, we plot the volume-weighted flow speed distribution for Parker and Bentheimer samples in Supplementary Figures S3(b) and S3(d), respectively. Both distributions are bimodal, however, with varying peak positions and relative weights. While the first peak occurs at negligible flow speeds, of the order of pm/s, the second peak exhibits flow speeds of the order of µm/s. In Parker, there is a larger fraction of the fluid volume at rest, leading to a small volume-weighted average flow speed of 2 µm/s. In contrast, Bentheimer has a much higher volume-weighted average flow speed of 23 µm/s. The comparison of flow speed distributions reveals that the connection between porosity and permeability is not straightforward: the near-zero-velocity peak represents the volume fraction of the porous medium that, despite being fully connected, does not contribute significantly to permeability.

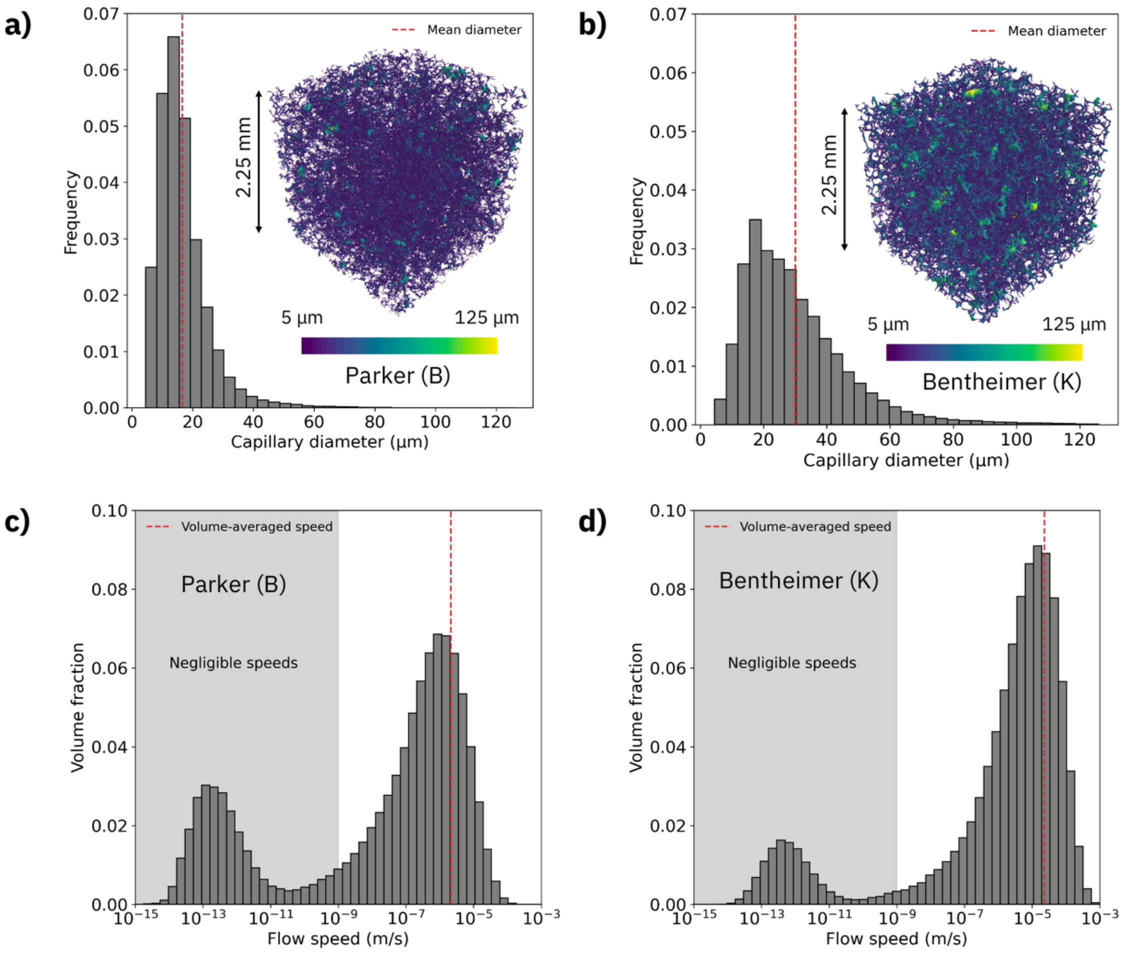

Supplementary Figure S3: Capillary diameter and flow speed distribution, respectively, obtained by CNM. Capillary diameter distribution of (a) Parker and (b) Bentheimer rock samples investigated in this study, together with visualizations of their capillary network as inset graphs. Darker colors (purple) represent smaller capillary diameters while lighter colors (yellow) represent larger diameters. (c), (d) Volume-weighted flow speed distribution of same the samples shown in (a) and (b), respectively.

In Supplementary Figure S4, we plot visual maps of the functional dependence of flow speed on capillary diameter obtained for four representative rock samples. All capillary diameter distributions are unimodal, while the flow speed distributions are bimodal. A weak correlation exists between the positions of the peaks, such that the peak at lower flow speed is more prominent for lower diameters and the peak at higher flow speeds is more prominent for higher diameters. Nevertheless, for any given diameter we observe both slower and faster flow speed components in the graphs.

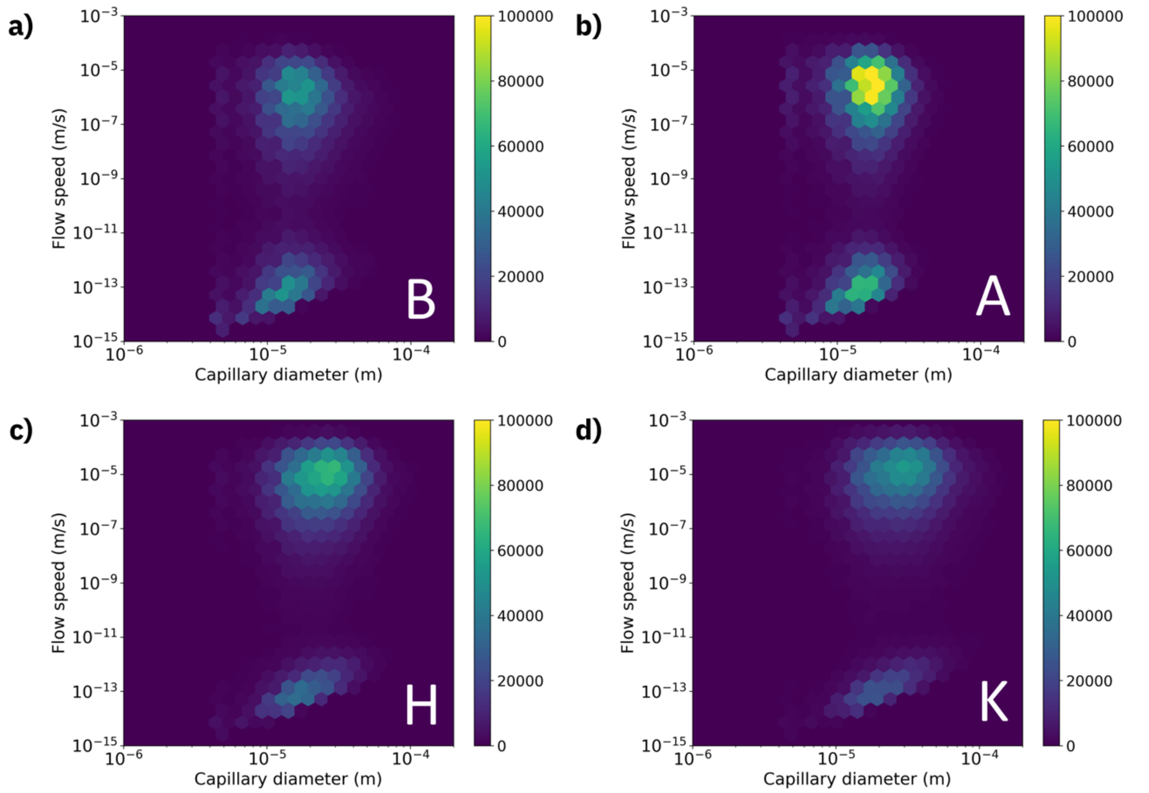

Supplementary Figure S4: Visualizing flow speeds versus capillary diameters for representative rock samples. 2D-false-color plots depicting (a) the least porous, (b) the least permeable, (c) the most porous and (d) the most permeable sample of this study, respectively.

Supplementary References

SR1. Bitter, I., Kaufman, A. E. & Sato, M. Penalized-Distance Volumetric Skeleton Algorithm. *IEEE Trans. Vis. Comput. Graph.* **7**, 195–206 (2001).

SR2. Zhou, Y., Kaufman, A. & Toga, A. W. Three-Dimensional Skeleton and Centerline Generation Based on an Approximate Minimum Distance Field. *Vis. Comput.* **14**, 303–314 (1998).

SR3. Tarjan, R. Depth-First Search and Linear Graph Algorithms. *SIAM J. Comput.* **1**, 146–160 (1972).

SR4. Dijkstra, E. W. A Note on Two Problems in Connexion With Graphs. *Numer. Math.* **1**, 269–271 (1959).
